# Brain Perivascular Macrophages Do Not Mediate Interleukin-1-Induced Sickness Behavior in Rats

**DOI:** 10.3390/ph14101030

**Published:** 2021-10-11

**Authors:** Léa Chaskiel, Robert Dantzer, Jan Pieter Konsman

**Affiliations:** 1 UMR CNRS 5287 Aquitaine Institute for Integrative and Cognitive Neuroscience, University of Bordeaux, 33076 Bordeaux, France; lea.chaskiel@gmail.com; 2 Department of Symptom Research, MD Anderson Cancer Center, The University of Texas, Houston, TX 770030, USA; RDantzer@mdanderson.org

**Keywords:** brain perivascular macrophages, food intake, interleukin-1, locomotor activity, social interaction

## Abstract

Sickness behavior, characterized by on overall reduction in behavioral activity, is commonly observed after bacterial infection. Sickness behavior can also be induced by the peripheral administration of Gram-negative bacterial lipopolysaccharide (LPS) or interleukin-1beta (IL-1β), a pro-inflammatory cytokine released by LPS-activated macrophages. In addition to the microglia, the brain contains perivascular macrophages, which express the IL-1 type 1 receptor (IL-1R1). In the present study, we assessed the role of brain perivascular macrophages in mediating IL-1β-induced sickness behavior in rats. To do so, we used intracerebroventricular (icv) administration of an IL-1β-saporin conjugate, known to eliminate IL-R1-expressing brain cells, prior to systemic or central IL-1β injection. Icv IL-1β-saporin administration resulted in a reduction in brain perivascular macrophages, without altering subsequent icv or ip IL-1β-induced reductions in food intake, locomotor activity, and social interactions. In conclusion, the present work shows that icv IL-1β-saporin administration is an efficient way to target brain perivascular macrophages, and to determine whether these cells are involved in IL-1β-induced sickness behavior.

## 1. Introduction

Sickness behavior, consisting of reductions in food intake (hypophagia), locomotor activity, and social interactions, is commonly associated with bacterial infection [1,2,3]. Pro-inflammatory cytokines, such as interleukin-1 (IL-1), produced by activated immune cells in response to bacterial lipopolysaccharides (LPS) [4,5,6], also reduce food intake, activity, and social interactions, and mediate sickness behavior [7,8,9,10,11,12]. Over the past decades, prostaglandin synthesis at the blood–brain barrier (BBB), activation of the vagus nerve, and the central transport or synthesis of IL-1 have been proposed to take part in the immune-to-brain signaling underlying sickness-associated changes in behavior [13]. In accordance with the first hypothesis, the endothelial-targeted knockdown of the IL-1 type 1 receptor (IL-1R1) in mice has been found to abrogate sickness behavior after intravenous (iv), but not after intraperitoneal (ip), administration of IL-1β [14]. However, we recently showed that IL-1R-expressing neurons in the arcuate hypothalamus of rats play a role in hypophagia after ip IL-1β administration [15], thus corroborating the hypothesis that IL-1β action in the brain is important for sickness behavior.

However, given that the arcuate nucleus seems much more accessible to high-molecular-weight components circulating in the blood, rather than to those present in the cerebrospinal fluid [16,17], IL-1-receptors in this structure may be a preferential target for the action of systemic IL-1β, instead of IL-1β produced in the brain. In addition to neurons, rodent brain endothelial cells and macrophages, and, to a lesser extent, astrocytes, express IL-1R1 [14,18,19]. Interestingly, peripherally administered LPS induces IL-1β immunoreactivity in the macrophages of brain circumventricular organs, such as the organum vasculosum lamina terminalis (OVLT) and choroid plexus [20,21], as well as bioactive IL-1β in the rat ventricular cerebrospinal fluid [22]. Moreover, intracerebroventricular (icv) administration of IL-1β rapidly spreads through the perivascular spaces and induces nuclear translocation of the transcription factor NF-κb in perivascular cells [23,24], in addition to sickness behavior [25,26,27]. Furthermore, circulating IL-1β has been proposed to act on rat brain perivascular macrophages to induce the prostaglandin-synthesizing enzyme cyclooxygenase-2 [28]. Thus, these findings raise the question as to whether central and/or peripheral IL-1β acts on brain perivascular macrophages to induce sickness behavior.

The cell types mediating the behavioral effects of IL-1 have been mostly studied using different promotor-specific Cre-Lox recombinant mice. During mouse development, the angiopoietin-1 receptor promotor Tie-2 is highly expressed in brain endothelial cells [29], but also in myeloid precursors that give rise to brain F4/80-positive cells [30], while expression of the fractalkine receptor promoter Cx3cr1 occurs in the microglia and, to a lesser extent, in brain macrophages, which both express Iba-1 [31,32]. In contrast, the thyroid hormone transporter and solute carrier organic anion transporter family member 1C1 (Slco1c1) promoter is expressed in rodent brain parenchymal endothelial cells, tanycytes, choroid plexus cuboid cells, and some cortical and hippocampal neurons [33,34,35]. Thus, it has been shown that Tie-2-dependent, but not Cx3cr1- or Slco1c1-dependent, genetic deficiency for IL-R1, or the IL-R1-associated signaling protein Myd88, abrogates sickness behavior induced by icv, but not ip, IL-1β [14,19,24]. Given the still-debated expression of Cx3cr1 in brain macrophages [31,32,36,37], these findings can be interpreted to suggest that IL-1R1 signaling in the fenestrated capillaries of brain circumventricular organs and choroid plexus [24], or in brain macrophages, plays a role in IL-1β-induced sickness behavior.

The present experiments were designed to test the hypothesis that IL-1R1-expressing brain perivascular macrophages mediate IL-1β-induced sickness behavior. Brain perivascular macrophages are a distinct population of myeloid cells that differ from microglia in their developmental origin, location, and activity profile [32,37,38]. To specifically target IL-1R1-expressing brain perivascular macrophages, we used icv administration of an IL-1ß-saporin conjugate that we recently validated to lesion brain IL-1 receptor-expressing cells [15]. Saporin is a ribosomal-inactivating protein [39], which is non-toxic outside the cells, but can enter the cell if coupled to a ligand that is internalized once bound to its receptor [40,41]. Given that icv IL-1 preferentially spreads along the perivascular spaces [23], the administration of IL-1ß-saporin via this route is expected to selectively eliminate IL-R1-expressing brain perivascular macrophages and, therefore, allow the role of these cells in IL-1β-induced sickness behavior to be established.

## 2. Results

### 2.1. Ip IL-1 β-Alexa Conjugate Decreased Food Intake

A one-way ANOVA on food intake after ip injection showed that 60 µg/kg of mouse (m)IL-1β-Alexa-488 significantly reduced cumulated food intake 2, 3.5 and 5 h later, as compared to the administration of a vehicle (F(1,14) = 5.94, *p* < 0.05; F(1,12) = 8.22, *p* < 0.05; F(1,10) = 5.92, *p* < 0.05, respectively (data not shown)), thus indicating that the conjugation reaction did not affect IL-1β’s bioactivity.

### 2.2. Icv IL-1β-Saporin Conjugate Eliminated Brain Perivascular Macrophages, but Not Endothelial Cells

**Figure 1:**
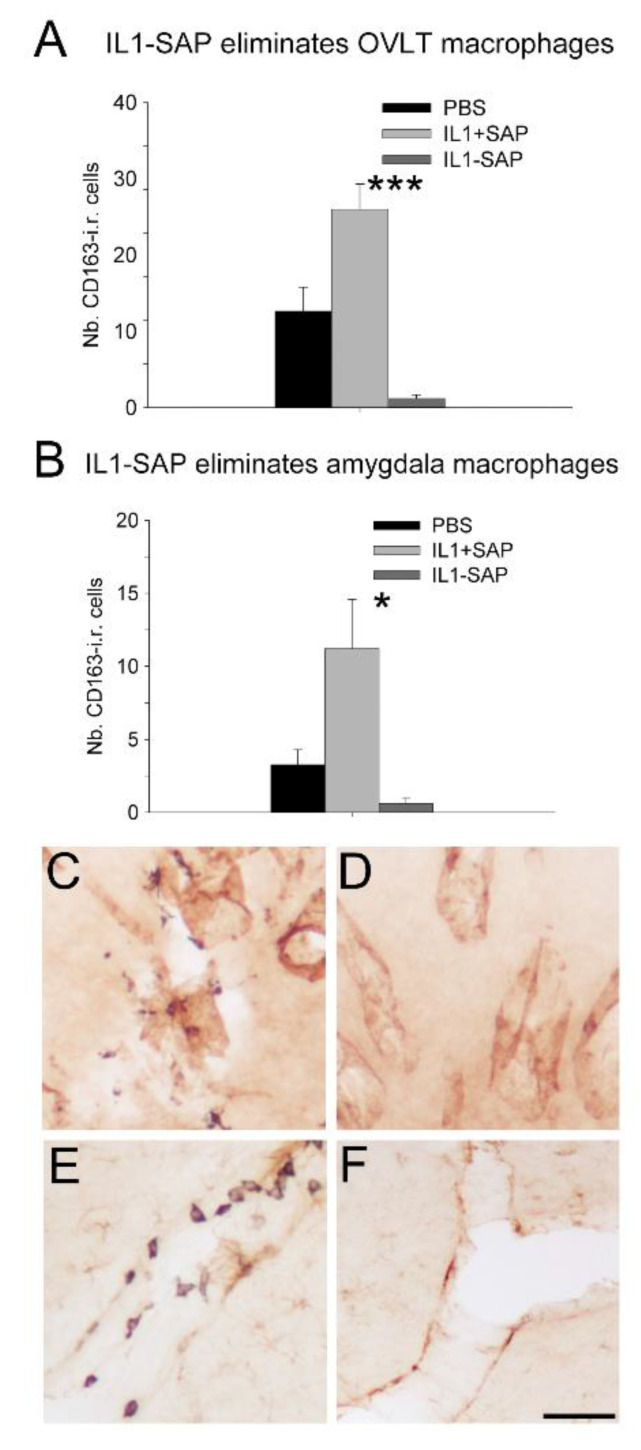
Effects of intracerebroventricular IL-1ß-saporin conjugate administration on brain perivascular cells in the organum vasculosum laminae terminalis (OVLT; **A**,**C**,**D**) and amygdala (**B**,**E**,**F**). In these structures, the IL-1ß-saporin conjugate reduced the number of CD163 immunoreactive (CD163 i.r.) cells (**A**–**F**) without affecting labelling for the endothelial cell marker von Willebrand factor (**C**,**D**) and the astrocyte marker glial fibrillary acidic protein (**E**,**F**). Brown labeling indicates von Willebrand factor (**C**,**D**) and glial fibrillary acidic protein (**E**,**F**) and black staining CD163. * *p* < 0.05; *** *p* < 0.001. IL1-SAP compared to IL1 + SAP group. IL1: interleukin-1ß; IL1-SAP: interleukin-1ß conjugated to saporin; IL1 + SAP: interleukin-1ß not conjugated to saporin; PBS: phosphate-buffered saline; SAP: saporin. Group sizes: *n* = 5–9. Scale bar represents 100 µm.

After semi-quantitative immunohistochemistry, one-way ANOVA on the number of CD163-positive cells in the OVLT, where many perivascular macrophages are concentrated, revealed a significant effect of icv pretreatment (F(2,26) = 19.0, *p* < 0.001). Post hoc tests showed that mIL-1β-saporin conjugate administration reduced the number of CD163-positive cells in the OVLT, as compared to PBS or mIL-1β + saporin (not conjugated) administration (*p* < 0.05 and *p* < 0.001, respectively; Figure 1A,C). Similar analyses on blood vessels within the amygdala indicated a significant effect of icv pretreatment on the number of CD163-positive cells (F(2,27) = 6.54, *p* < 0.01). Post hoc tests then revealed that the administration of mIL-1β + saporin induced an increase in CD163-positive cells in the amygdala, as compared to that of PBS (*p* < 0.05), but also indicated that the icv administration of the mIL-1β-saporin conjugate reduced the number of perivascular macrophages, compared to mIL-1β + saporin administration (*p* < 0.05. Figure 1B). Finally, and according to one-way ANOVA, icv pretreatment had a significant effect on the surface of CD163-positive cells, relative to the surface of vessels located between the cortex and the striatum (F(2,27) = 8.75, *p* < 0.001). Subsequent post hoc tests showed that the mIL-1β-saporin conjugate decreased the relative surface of CD163-positive cells, compared to PBS or mIL-1β + saporin (*p* < 0.05 and *p* < 0.001, respectively; data not shown). At those locations where icv administration of the mIL-1β-saporin conjugate reduced the number of perivascular macrophages, no major differences in GFAP and vWF immunoreactivity were observed between the icv pretreatments (Figure 1C,D).

### 2.3. Icv IL-1β-Saporin Conjugate Did Not Alter Behavioral Responses to Subsequent IL-1β Injection

**Figure 2:**
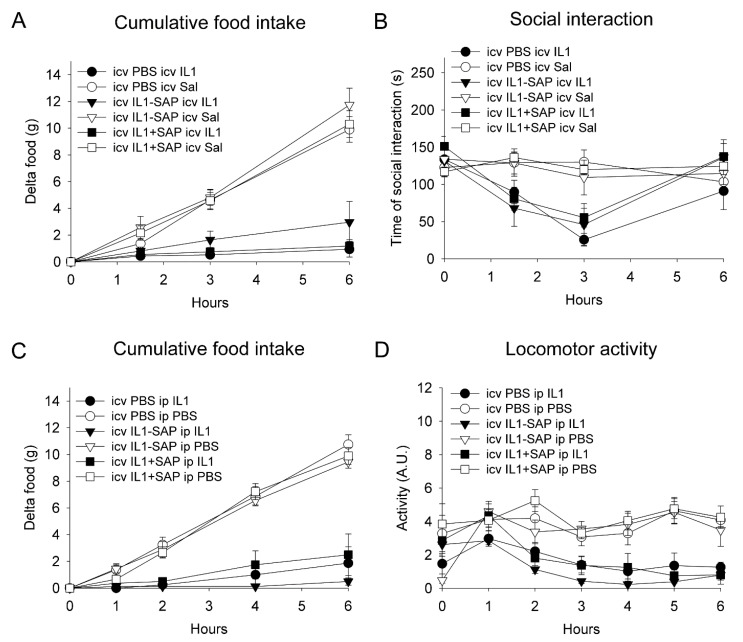
Effects of intracerebroventricular IL-1ß-saporin conjugate administration on subsequent IL-1β-induced sickness behavior. Intracerebroventricular pre-treatment with IL-1β-saporin conjugate did not affect the reduction in food intake and social interaction after intracerebroventricular IL-1ß administration (**A**,**B**). Intracerebroventricular administration of IL-1β-saporin conjugate did not affect the reduction in food intake and locomotor activity to subsequent intraperitoneal IL-1ß injection (**C**,**D**). IL1: interleukin-1ß; IL1-SAP: interleukin-1ß conjugated to saporin; IL1 + SAP: interleukin-1ß not conjugated to saporin; PBS: phosphate-buffered saline; Sal: saline; SAP: saporin. Group sizes: *n* = 5–9.

A two-way ANOVA on cumulative food intake, over six hours after icv rat recombinant (rr)IL-1β or saline administration, five days after pre-treatment, indicated a significant reduction in icv rrIL-1β-injected rats as compared to vehicle-treated animals (F(1,29) = 112, *p* < 0.001), with no significant effect of the pre-treatment or interaction between the pre-treatment and treatment (Figure 2A). Similarly, a three-way repeated measures ANOVA on social exploration, 1.5, 3 and 6 h after icv rrIL-1β or saline administration (treatment), showed that rrIL-1β induced a significant decrease (F(1,29) = 16.5, *p* < 0.001), without any significant effect of the pre-treatment (Figure 2B).

A two-way ANOVA on cumulative food intake, over six hours after ip rrIL-1β or saline administration, five days after pre-treatment, indicated a significant reduction in icv rrIL-1β-injected rats as compared to vehicle-treated animals (F(1,41) = 31.1, *p* < 0.001), with no significant effect of the pre-treatment or interaction between the pre-treatment and treatment (Figure 2C). Similarly, a three-way repeated measures ANOVA on locomotor activity, during the first six hours after ip rrIL-1β or saline administration (treatment), showed that rrIL-1β induced a significant overall decrease (F(1,34) = 77.4, *p* < 0.001), without a significant effect of the pre-treatment or interaction between the treatment and pre-treatment (Figure 2D).

## 3. Discussion

The aim of the present experiments was to determine the role of IL-1R1-expressing brain perivascular macrophages in mediating IL-1β-induced sickness behavior, using an IL-1R1-saponin conjugate to specifically eliminate cells containing IL-1R1 [15]. The administration of this conjugate reduced the number of CD163-positive brain perivascular macrophages, known to express IL-1R1 [18], without altering the immunoreactivity for endothelial cells or astrocytes, but did not affect sickness behavior in response to either icv or ip administration of IL-1β. These findings are important because they allow, for the first time, IL-1R1-expressing brain perivascular macrophages to be targeted in a more direct way, as compared to the use of promotor-specific Cre-Lox recombinant mice. In addition, they indicate that perivascular macrophages do not play an important role in the behavioral effects of exogenously administered IL-1β.

In experimental life sciences, it is of the utmost importance to intervene on a target causal candidate in different ways. Thus, it has been argued that repeating the same experiments is not enough to promote robust research, but that, in addition, different lines of evidence are required to mitigate the biases associated with findings obtained using a particular approach to address a research question [42]. When it comes to the question of which cell types mediate the behavioral effects of IL-1, the vast majority of recent studies have employed different promotor-specific Cre-Lox recombinant mice. One of the major limitations of such approaches is the specificity of the promotors used. Indeed, specific promotors may not yet be widely available for all cell types, and the specificity of promotors that have already been used may be less than initially presumed. In particular, there is an ongoing discussion about the expression of the Cx3cr1 promotor in brain cells. While some authors have presented Cx3cr1-Cre-Lox recombinant mice as lacking expression of a gene of interest only in the microglia, there is increasing evidence to suggest that this promotor is expressed by brain macrophages and, in some conditions, by neurons [31,32,36,37,38,43]. Similarly, the Ttie-2 promotor Tie-2 is highly expressed in brain endothelial cells [29], but has also been found in myeloid precursors that give rise to brain cells expressing F4/80 [30], which is one of the markers expressed by perivascular macrophages [32,37,38]. Thus, the currently widely employed promotor-specific Cre-Lox recombinant mice do not seem to be adequate to address the role of brain perivascular macrophages in the behavioral effects of IL-1β. Another important limitation of the studies published thus far, addressing the brain cell types mediating IL-1β’s effects on behavior, is that they have all been conducted in mice.

The role of brain perivascular macrophages in the effects of IL-1β on behavior was, therefore, studied in rats using non-genetically modified animals. One of the traditional ways of targeting these cells is the icv administration of liposomes containing clodronate. This treatment results in phagocytosis of these particles, by perivascular macrophages, and subsequent uptake of the apoptosis-inducing drug clodronate [44]. Although the initial article based on this technique reported no effect of such treatment on the microglia [44], recent work indicates that the icv administration of liposomes containing clodronate can also reduce the number of microglia in adult rodent brains [45,46]. Since microglia do not express IL-1R1 under basal conditions [18,19], icv administration of the IL-1ß-saporin conjugate that was previously shown to specifically lesion brain IL-1 receptor-expressing neurons in the hippocampus [15] appears to be an efficient way to eliminate brain perivascular macrophages without affecting the microglia. However, since icv-administered IL-1 preferentially spreads along the brain perivascular spaces [23], perivascular cell types, other than macrophages, known to express IL-R1, such as endothelial cells and astrocytes [18,19], may also be a target of icv IL-1ß-saporin. This is the reason why, in our previous and present work, the effect of IL-1ß-saporin administration on brain endothelial cells and astrocytes was studied. Importantly, we have shown that immunoreactivity for the endothelial cell marker von Willebrand factor, and that for the astrocyte marker glial fibrillary acidic protein, was not affected by intracerebral or icv administration of the IL-1ß-saporin conjugate [15]. The absence of an effect of IL-1ß-saporin on these cell types may be explained by postulating that IL-R1s are neither expressed on the abluminal side of brain endothelial cells nor on astrocytic end feet. Indeed, the only rat brain cell types that were affected by the IL-1ß-saporin conjugate were the hippocampal and arcuate hypothalamic neurons after local microinjection [15], and perivascular macrophages after icv administration (present work). Icv administration of IL-1ß-saporin is, therefore, an important experimental tool to study the contribution of IL-R1-expressing brain perivascular macrophages to mediating IL-1β-induced sickness behavior.

Because of their location in the perivascular spaces, and their close contact with the abluminal site of brain endothelial cells, perivascular macrophages have long been thought to connect the immune and nervous systems [47,48,49]. Beyond their roles as scavenger phagocytes in neuropathologies, these cells have been shown to play a role in hypertension-associated neurovascular and cognitive dysfunction, activation of the paraventricular nucleus of the hypothalamus, and corticosterone and noradrenaline release after systemic IL-1β administration or myocardial infarction in rodents [36]. However, their role in mediating IL-1β-induced sickness behavior has not been studied directly. Since this pro-inflammatory cytokine can be detected peripherally as well as in the brain in animal models of systemic inflammation [21,50], IL-1β was injected either ip or icv after icv administration of IL-1ß-saporin conjugate to study the role of brain macrophages in sickness behavior. However, even though icv IL-1β-saporin administration significantly reduced the number of brain perivascular macrophages, it did not alter subsequent icv or ip IL-1ß-induced reduced food intake, locomotor activity, and social interactions. These findings indicate that IL-1R1-expressing brain perivascular macrophages do not mediate sickness behavior after intracerebroventricular IL-1β injection. This lack of effect could theoretically be due to the fact that an in vivo behavioral assessment was performed at five days after administration, several days before the post-mortem histological examination. However, this is rather unlikely, as other populations of macrophages have been shown to be reduced within one week after an injection of saporin conjugates [51,52,53]. Moreover, increasing the interval between IL-1β-saporin conjugate administration and subsequent IL-1ß injection to thirteen days did not affect the sickness behavior [15]. Finally, IL-R1s in the rat arcuate hypothalamus mediate in part hypohagia after systemic IL-1ß injection, since the local administration of IL-1β-saporin into this brain structure mitigated, without preventing, ip IL-1ß-induced reduced food intake [15]. Therefore, the most likely explanation for our finding is that brain perivascular macrophages do not play a role in IL-1ß-induced sickness behavior.

## 4. Materials and Methods

### 4.1. Animals

Thirty-three and fifty male sexually naïve Wistar Han IGS: [Crl:WI(Han)] rats that weighed 175–225 g upon arrival (Charles River, L’Arbresle, France) were housed five per polypropylene cage (30 × 45 × 19 cm) containing corncob bedding (UAR, Epinay-sur-Orge, France) and left undisturbed for fourteen days in a room with controlled temperature (23 ± 1 °C) and light (12/12 h light–dark cycle) conditions and ad libitum access to food (Extralabo, Provins, France) and water. Twenty-one- to thirty-day-old juvenile animals of the same strain were used as social stimuli and housed in groups of ten in a similarly controlled room. Studies were carried out in accordance with the INRA quality reference system (www.international.inra.fr/content/download/947/11111/file/requirements.pdf accessed on 1 September 2014), approved by the Aquitaine Poitou-Charente regional ethical committee on animal experiments (AP 1 March 2004) and followed European recommendations on animal research (European Council Directive of 24 November 1986 (86/609/EEC) and European Parliament and Council Directive of 22 September 2010 (2010/63/UE)).

### 4.2. Biologically Active Molecules

Purified rat recombinant interleukin-1beta (rrIL-1ß; biological activity: 317 IU/mg, NIBSC, Potters Bar, UK) was dissolved in sterile phosphate-buffered saline (PBS), as previously described [54], and used for intracerebroventricular (icv) injections. Non-targeted saporin, a ribosome-inactivating protein from Saponaria officinalis (Advanced Targeting Systems, San Diego, CA, USA), dissolved in sterile PBS, was used for the conjugation of saporin to IL-1ß and as a control for treatment with this conjugate (see below). Since rat IL-1ß contains four cysteine residues, coupling to saporin would be likely to result in a heterogeneous molecule with variable toxicity. However, mouse, similar to human, IL-1ß only contains one cysteine residue and is bioactive in rats [55,56,57]. Furthermore, this cysteine residue does not affect biological activity [58], which allowed this residue to be changed to serine by site-directed mutagenesis to result in mouse [S71]IL-1ß. Next, a new cysteine was introduced by changing the C-terminal serine to cysteine resulting in [S71,C152]mIL-1ß. This molecule was used as starting material for conjugation to Alexa-488, a chemically modified fluorescein molecule, or to saporin, and as a supplementary control for the effects of the conjugate. The molecules were first derivatized by N-succinimidyl-3-(2-pyridyl) dithiopropionate (SPDP; Pierce, Perbio Science, Brebières France) at room temperature for 24 h and subsequently coupled to the cysteine residue of mouse [S71,C152]IL-1ß for another 24 h at room temperature. The molar ratio of Alexa-488 or saporin to mouse [S71,C152]IL-1ß was 1:1. The coupling reaction between [C71,C152]mIL-1ß and Alexa-488 resulted in a single peak of emission centered around 494 nm (not shown). The coupling reaction between [C71,C152]mIL-1ß and saporin yielded a conjugate molecule migrating at the expected size on an electrophoresis gel [15].

### 4.3. Sickness Behavior Testing after Intraperitoneal Injection of Interleukin-1β-Alexa-488

To assure that mouse [S71,C152]IL-1β conjugates were biologically active and induced sickness behavior, rats were given an ip injection of 60 µg/kg [S71,C152]mIL-1β-Alexa-488 or saline two hours before the start of the dark phase. Food intake was monitored by weighing food pellets in ruffs just before and 2, 3.5 and 5 h after injection on a high-precision balance. Even though rats show their food intake peak during the first hours of the dark phase if they have ad libitum access to food, each animal has its own pattern that is repeated everyday [59]. Cumulative food intake over 5 h was therefore chosen for data presentation and analysis. One week after the first injection, treatments were reversed according to a Latin square design.

### 4.4. Intracerebroventricular Injection of Interleukin-1β ± Saporin

Rats were first anesthetized through an ip injection (1 mL/kg) of ketamine (61 mg/kg) and xylazine (9 mg/kg) and secured in a stereotaxic apparatus. A midline incision was made and skin flaps were pulled aside.

In thirty-three animals, a stainless-steel 7 mm long guide cannula (23 Gauge) was placed above the lateral brain ventricle for subsequent icv administration of [S71,C152] mouse interleukin-1β ± saporin or vehicle and ultimately ip rat interleukin-1β or vehicle (see below). This guide cannula was lowered unilaterally 3.2 mm below the skull surface (2.2 mm below the dura mater) through a hole drilled in the skull 0.6 mm posterior to bregma and 1.5 mm lateral to the midline. Coordinates were chosen to place the tip of this guide cannula 1 mm above the roof of the lateral ventricle. Guide cannulas were permanently connected to the skull with dental cement and screws. The head wound was closed by suturing skin flaps and rats were allowed a two-week recovery period before icv injections and behavioral testing. For icv injections into the lateral ventricle, a 30-gauge 8 mm long injection cannula was lowered through the guide cannula and connected to a 10 µL Hamilton syringe with plastic tubing. Ten microliters of the following solutions were infused to deliver (1) 1.75 μg conjugated [S71,C152]mIL-1β-saporin dissolved in phosphate-buffered saline (PBS; [S71,C152]mIL-1ß-saporin; *n* = 12), (2) equivalent amounts of unconjugated saporin and [S71,C152]mIL-1ß dissolved in PBS ([S71,C152]mIL-1ß + saporin; *n* = 9), or (3) PBS (PBS; *n* = 12) to hand-held animals. The injection device was left in place for another thirty seconds to allow diffusion of chemicals or vehicle and then retracted. These rats were subsequently used to study the effects of these pretreatments on icv IL-1ß-induced sickness behavior (see below).

The doses of [S71,C152]mIL-1β-saporin conjugate used were based on the finding that 6–600 ng of mouse IL-1β administered icv reduces food intake in the rat [55], bearing in mind that only 40% (17 kDa/42 kDa), about 700 ng, of the weight of the IL-1β-saporin construct corresponds to IL-1β. A 10-fold excess of the IL-1β-saporin construct was infused icv relative to the amount of rrIL-1β administered five days later via the same guide cannula (see below).

In fifty animals, a glass micropipette with a tip diameter of 100–120 µm was placed 8.5 mm below the midline dural surface, 1.8 mm posterior to bregma, for third brain ventricle administration of [S71,C152] mouse interleukin-1β ± saporin or vehicle. This micropipette was connected to a 10 µL Hamilton syringe with plastic tubing with the aid of which five µL of solution was infused to administer the following: (1) 875 ng conjugated [S71,C152]mIL-1β-saporin dissolved in PBS ([S71,C152]mIL-1ß-saporin; *n* = 17), (2) equivalent amounts of unconjugated saporin (330 ng) and [S71,C152]mIL-1ß (544 ng) dissolved in PBS ([S71,C152]mIL-1ß + saporin; *n* = 17), or (3) PBS (PBS; *n* = 16). These rats were subsequently used to study the effects of these pretreatments on ip IL-1ß-induced sickness behavior (see below).

After the head wound was closed by suturing skin flaps, a telemetric transmitter (TA10TA-F40, Data Sciences, St. Paul, MN, USA) was inserted into the peritoneal cavity of each rat through a 2 cm incision in the linea alba. The abdominal wall and skin were then sutured. A ten day period was given to the animals to recover from surgery and to allow enough time for saporin to provoke lesions. Food intake and body weight were measured daily from the day before surgery until the end of the experiments.

### 4.5. Sickness Behavior Assessment after Intracerebroventricular Interleukin-1β Injection

Since conjugated saporin constructs can kill 66–75% of cells, including macrophages, within 5 days [53,60,61], a challenge with icv rr IL-1β or saline was given five days after icv pretreatment of IL-1ß ± saporin or PBS. Thus, during the early dark phase, rats were administered icv with either 70 ng of rrIL-1ß dissolved in 2 µL of saline or vehicle at a rate of 0.5 µL every thirty seconds. Social interaction with a juvenile rat of the same strain and food intake were measured (see below) 1.5, 3 and 6 h later as measures of sickness behavior. Social behavioral testing took place in the home cage of the test animal as previously described [15,50]. A trained observer, blind to the treatment given, scored the duration of social exploration of the test rat directed towards the juvenile rat from a video screen fed by a camera placed over the home cage using pre-set keys on the keyboard of a microcomputer. Social exploration consisted mainly of ano-genital sniffing, but also included nosing, sniffing of body parts, and grooming behaviors. Food intake and body weight changes were obtained by measuring weight of food pellets in ruffs and rats just before and 1.5, 3 and 6 h after injection on a high-precision balance. Cumulative food intake and changes in body weight over 6 h were chosen for data presentation and analysis.

### 4.6. Sickness Behavior Assessment after Intraperitoneal Interleukin-1β Injection

Ten days after third brain ventricle administration, half of the animals of each experimental group received an intraperitoneal injection (1 mL/kg) of sterile PBS and the other half an intraperitoneal injection of IL-1β (30 µg/kg) one hour before the lights went off. Horizontal locomotor activity and food intake were used as measures of sickness behavior. Food intake was measured at the time of ip injection and 1, 2, 4, 6 h after injection. Locomotor activity was monitored every 10 min for 10 s with the implanted telemetry devices based on the changes in power of the transmitted signal due to the transmitter position changes and fed into a computer-based data acquisition system (Dataquest IV, Data Sciences, St. Paul, MN, USA).

### 4.7. Brain Tissue Preparation

Twenty-four hours after the last injections, animals were anesthetized with pentobarbital (150 mg/kg) and perfused transcardially with 0.9% NaCl followed by 4% paraformaldehyde (pH 7.4). Fixed brains were removed from the skull and placed for another 4 h in the same fixative before being transferred to 30% sucrose and stored at 4 °C for 48 h. Brains were subsequently frozen and stored at −80 °C. Forty-micrometer brain sections were cut on a cryostat and stored in a cryoprotective solution (20% glycerol, 30% ethylene glycol in PBS, 0.1 M, pH 7.4) at −20 °C.

### 4.8. Immunohistochemistry

To determine to what extent icv administration of IL-1β-saporin conjugate affected brain macrophages, astrocytes and endothelial cells, immunohistochemistry was employed to detect the macrophage-specific marker CD163, the astrocyte-specific marker glial fibrillary acidic protein (GFAP) and the endothelial-specific marker von Willebrand factor (vWF), respectively. Immunohistochemistry was performed as previously described [15,50]. Briefly, free-floating sections were washed four times in 0.1 M PBS (pH 7.4). Non-specific binding sites were blocked by a 45-minute incubation in PBS containing 0.3% Triton X-100 and 1.0% bovine serum albumin (BSA). The first antibody was subsequently diluted in the same buffer and added to sections overnight at room temperature. Commercially available antisera raised against CD163 (Serotec, mouse anti-rat CD163, MCA342R), GFAP (rabbit anti-GFAP, Sigma, 63893, Sigma-Aldrich, St-Fallavier, France), and vWF (rabbit anti-vWF, ab6994, Abcam, Paris, France), all diluted 1:1000 were used. These antisera have been well characterized according to the Journal of Comparative Neurology antibody database available at antibodyregistry.org (IDs for CD163, GFAP, and vWF antibodies were AB_321966, AB_477010, and AB_305689, respectively). After four rinses in PBS, sections were treated for 30 min with 0.3% (*v/v*) hydrogen peroxide to block endogenous peroxidases followed by additional rinses in PBS. Sections were then incubated for 2 h with biotinylated antisera raised against mouse or rabbit IgGs (Vector Laboratories, Burlingame, CA, USA) diluted 1:1000 in PBS containing 0.3% Triton X-100 and 1% BSA. After four washes in PBS, sections were incubated for 2 h with a complex of avidin and biotinylated peroxidase (Vector Laboratories, Burlingame, CA, USA) diluted 1:500 in PBS. Finally, sections were transferred to a sodium acetate buffer and stained using diaminobenzidine as a chromogen in the presence of Ni ions, thus yielding a dark grey to black precipitate. In the case of double-labeling immunohistochemistry, CD163 immunoreactivity was revealed following this staining procedure, after which sections were incubated with GFAP or vWF antibodies. The binding of these antibodies was then revealed following the same protocol, except that staining occurred in the absence of Ni ions and thus resulted in brown labeling.

### 4.9. Microscopy

Stained sections were examined with a light microscope (Nikon Eclipse, Nikon France or Leica DM5500B, Leica Microsystems, Nanterre, France) and images were captured using a high-resolution CCD video camera and fed into a computer. To quantify CD163-immunoreactive cells, image processing was performed using Image J (http://imagej.nih.gov/ij/ accessed on 1 September 2021) on grey images by defining brightness and surface area above which labeling was to be taken into account. Once established, these parameters remained unchanged. CD163-positive cells were thus quantified along vessels of the organum vasculosum laminae terminalis (OVLT), the amygdala and between cortex and striatum in at least three sections. Given (1) that cerebral arteries and veins almost never run in parallel in rats [62], (2) that arteries and arterioles stain for alkaline phosphatase, an arterial and arteriole cell marker [18], and (3) that more peri-arteriole perivascular macrophages than peri-venule perivascular macrophages are found in rodent brain [38,63,64], these latter vessels were considered to correspond to the anterior choroid artery and posterior striate artery, respectively.

### 4.10. Data Presentation and Statistical Analysis

All data are reported as means ± standard error of the mean (SEM). Food intake after ip [S71,C152]mIL-1β-Alexa-488 conjugate or saline administration was analyzed with one-way ANOVA. Social interaction after icv [S71,C152]mIL-1β ± saporin or PBS (pre-treatment) was expressed as time that the test rat spent in exploration of the juvenile conspecific and compared using one-way repeated measures ANOVA. Two-way ANOVA was used to determine the effects of icv [S71,C152]mIL-1ß±saporin or PBS pretreatment on subsequent icv rrIL-1ß-induced changes in food intake. The effects of pre-treatment and treatment on time spent in social interactions and locomotor activity were compared using a three-way repeated measures ANOVA. Finally, the effect of icv pre-treatment with [S71,C152]mIL-1β ± saporin or PBS on the number ED2-positive cells was analyzed using one-way ANOVA. Significant effects were further analyzed by Newman–Keuls post hoc tests. When normality and equal variance criteria were not met, non-parametric Mann–Whitney tests or ANOVA on ranks were performed. In the latter case, significant effects were further analyzed by Dunn’s post hoc test. In all cases, a level of *p* < 0.05 was considered as statistically significant.

## 5. Conclusions

In conclusion, the present study confirms that IL-1β-saporin is a useful experimental tool to study the brain cell types mediating sickness behavior, and shows that rat brain perivascular macrophages are not involved in bringing about IL-1ß-induced sickness behavior. Further research will need to determine which cell types, beyond arcuate hypothalamic neurons, play a role in mediating features of sickness behavior after IL-1ß injection in rats.

## Data Availability

Data is contained within the article.
